# Different effects of the DRD4 genotype on intrinsic brain network connectivity strength in drug-naïve children with ADHD and healthy controls

**DOI:** 10.1007/s11682-021-00521-9

**Published:** 2021-08-18

**Authors:** Shuangli Chen, Andan Qian, Jiejie Tao, Ronghui Zhou, Chuqi Fu, Chuang Yang, Qingxia Lin, JieJie Zhou, Jiance Li, Xiaoqi Huang, Meihao Wang

**Affiliations:** 1 Department of Radiology, The First Affiliated Hospital of Wenzhou Medical University, Wenzhou, 325000 China; 2 Department of Mental Health, The First Affiliated Hospital of Wenzhou Medical University, Wenzhou, 325000 China; 3 Department of Radiology, Huaxi MR Research Center, West China Hospital of Sichuan University, Chengdu, 610041 Sichuan China

**Keywords:** ADHD, Dopamine D4 receptor gene (DRD4), Resting-state fMRI, Degree centrality (DC)

## Abstract

The dopamine D4 receptor gene (DRD4) has been consistently reported to be associated with attention-deficit/hyperactivity disorder (ADHD). Recent studies have linked DRD4 to functional connectivity among specific brain regions. The current study aimed to compare the effects of the DRD4 genotype on functional integrity in drug-naïve ADHD children and healthy children. Resting-state functional MRI images were acquired from 49 children with ADHD and 37 healthy controls (HCs). We investigated the effects of the 2-repeat allele of DRD4 on brain network connectivity in both groups using a parameter called the degree of centrality (DC), which indexes local functional relationships across the entire brain connectome. A voxel-wise two-way ANCOVA was performed to examine the diagnosis-by-genotype interactions on DC maps. Significant diagnosis-by-genotype interactions with DC were found in the temporal lobe, including the left inferior temporal gyrus (ITG) and bilateral middle temporal gyrus (MTG) (GRF corrected at voxel level p < 0.001 and cluster level p < 0.05, two-tailed). With the further subdivision of the DC network according to anatomical distance, additional brain regions with significant interactions were found in the long-range DC network, including the left superior parietal gyrus (SPG) and right middle frontal gyrus (MFG). The post-hoc pairwise analysis found that altered network centrality related to DRD4 differed according to diagnostic status (p < 0.05). This genetic imaging study suggests that the DRD4 genotype regulates the functional integration of brain networks in children with ADHD and HCs differently. This may have important implications for our understanding of the role of DRD4 in altering functional connectivity in ADHD subjects.

## Introduction

Attention-deficit/hyperactivity disorder (ADHD) is a prevalent and highly heritable childhood-onset neurodevelopmental disorder characterized by age-inappropriate levels of inattention, hyperactivity, and/or impulsivity (Apa, 2013). The etiology of ADHD is strongly influenced by genetic factors, as demonstrated by twin and adoption studies (Albayrak et al., 2008), with its heritability estimated at 70–90% (Larsson et al., 2014).

Most of the candidate genes associated with ADHD belong to the dopaminergic system (Gizer et al., 2009). Of the dopamine-related genes, the exon III 48 base pair variable number tandem repeats (VNTR) polymorphism of dopamine receptor D4 (DRD4), which encodes the third cytoplasmic loop (Oak et al., 2000), is most widely studied (Klein et al., 2017). Several meta-analyses have shown that the DRD4 7-repeat (7R) allele is mostly associated with ADHD (D. Li et al., 2006) and is rare in Asian populations. Instead, it has been reported that the 2-repeat (2R) allele of DRD4 is an ADHD-risk allele in the Asian population (Hong et al., 2018; Leung et al., 2005). Implications of the genotype DRD4 2R allele in predicting more severe symptoms and poor ADHD treatment outcomes also exist (Cheon et al., 2007). In terms of function, the 7R and 2R alleles exhibit similar biochemical properties; they have blunted responses to dopamine compared to 4-repeat (4R; (Asghari et al., 1995). As Wang et al. inferred, one possibility is that the role of the 7R allele in Western populations is equivalent to that of the 2R allele in Asian populations (Wang et al., 2004). An association between DRD4 and some certain behavioral traits has gained conflicting evidence. For example, previous neuropsychological works suggest there is a positive relationship between the 7R allele and problematic behaviors such as heavy drinking (Laucht et al., 2007) or better performance in attentional control (Sheese et al., 2007). Because DRD4 appears to be associated with both adverse and advantageous outcomes, some researchers have proposed that the 7R variant (or the 2R variant) of DRD4 functions as a plasticity allele, which is highly sensitive to environmental influence. However, the potential biobehavioral mechanisms are not well understood.

The pathway from a gene to its associated behavior is complex. Recently, increasing functional MRI (fMRI) studies have been conducted to bridge the gap between genotypical and behavioral correlates. However, genetic imaging studies related to the genotype DRD4 2R allele are scarce. Previous task-based fMRI studies have shown that the DRD4 gene can also influence brain activity in areas involved in executive function (Gehricke et al., 2015; Gilsbach et al., 2012) and reward processing (Camara et al., 2010). Nearly all available genetic imaging studies have focused on the DRD4 7R allele in Western populations. More recently, a case–control ADHD-relevant study was the first to report significant diagnostic and genotypic interaction effects of the DRD4 2R allele on regional brain function, indicating dysfunction in sustained and divided attention (Kim et al., 2018). Subsequently, other studies using functional connectivity (FC) or independent component analysis (ICA) showed that the 2R allele is also associated with various brain networks and widespread intrinsic neural circuits, such as the frontal-striatal-cerebellar loop or prefrontal cortex (PFC) circles (Qian, et al., 2018a, 2018b). The above-mentioned fMRI studies have explored regional spontaneous brain activity or have analyzed specific brain networks that rely upon methods including seed-based FC and ICA. However, these approaches do not directly show important functional network topological changes, such as how the whole brain functionally interacts during the resting state.

Degree centrality (DC) is a measure used in graph theory to investigate global connectivity by measuring the number of instantaneous functional connections (or correlations) between a given voxel (node) and the rest of the brain, rather than with specific nodes or networks (Zuo et al., 2012). Thus, it allows us to quantify the importance of a node to the rest of the brain. Nodes with a high degree centrality are deemed to be ‘hubs’. Therefore, DC offers a new conceptual framework for revealing the network biology of ADHD (Jiang et al., 2019; Zhou et al., 2019). These studies have shown that children with ADHD are involved in a connectivity-based pathophysiological process. Given the implication of the DRD4 gene in the pathophysiology of ADHD and the role of the 2R allele in Asian populations, we considered that the effect of the 2R allele on the whole-brain connectome should be investigated.

In the current study, we employed DC to explore more specific neural pathway within the whole-brain network under the effect of DRD4 gene. The final sample included 47 children with ADHD and 37 healthy controls who had not received any medication before. We hypothesized that the prefrontal lobe and temporo-limbic structures may be key brain regions linked to DRD4, for two reasons: (1) Using Reverse Transcription-Polymerase Chain Reaction (RT-PCR) technology, it has been found that DRD4 is relatively highly expressed in the above brain regions (Mulcrone & Kerwin, 1997). (2) Functional and structural abnormalities in the prefrontal lobe and temporo-limbic structures have been found in ADHD patients (Hoogman et al., 2019; Rubia et al., 2014) and have been associated with certain cognitive functions such as inhibitory control (Rubia, 2018).

## Materials and methods

### Subjects

Fifty-nine children with ADHD were recruited from the Mental Health Center of the First Affiliated Hospital of Wenzhou Medical University. Also, 38 healthy controls (HCs) were recruited from a local primary school and were matched for handedness and intelligence quotient (IQ). All participants were recruited between March 2012 and November 2014. Among them, 12 children with ADHD and 1 HC participant were excluded because of unqualified head movement (described in Sect. 2.4 *Head Motion*). The final analysis included 47 children with ADHD (15 2R and 32 non-2R) and 37 HC participants (16 2R and 21 non-2R). All participants were Han Chinese and right-handed.

All children with ADHD fulfilled the *Diagnostic and Statistical Manual of Mental Disorders, Fourth Revision* (DSM-IV) criteria according to two experienced clinical psychiatrists, C.Y. and Q.L., based on a semi-structured diagnostic interview and the *Kiddie Schedule for Affective Disorders and Schizophrenia for School-Age Children – Present and Lifetime Versions* (K-SADS-PL) (Kaufman et al., 1997). Among them, we excluded comorbid Axis I disorders, such as affective disorders and oppositional defiant disorder (ODD). Children who had a treatment history of methylphenidate (MPH) or atomoxetine and a full-scale IQ below 85 (as measured by the *Wechsler Abbreviated Scale of Intelligence*) were excluded. Other exclusion criteria were similar to those used in our previous study (Qian, et al., 2018a, 2018b). The healthy children were also screened by the same psychiatrists, according to the *Structured Clinical Interview* for DSM-IV, and the exclusion criteria were the same as those applied to the children with ADHD.

The *Conner’s Parent Symptom Questionnaire–Chinese, revised version *(Conners, 1999) was completed by all parents of the studied children to assess problematic behavior such as conduct and study problems. Additionally, all participants were asked to complete integrated visual and auditory continuous performance testing (IVA-CPT). The IVA-CPT is commonly used for the diagnosis of ADHD patients and reflects the function of attention and control (Tinius, 2003).

The protocol was reviewed and approved by the ethics committee of the First Affiliated Hospital of Wenzhou Medical University. Written informed consent was obtained from the guardians of all subjects. Table 1 summarizes the demographic and clinical characteristics of the participants.

**Table 1:** Demographics and clinical characteristics of the participants

Characteristics	ADHD		HC		p-values		
	2R (15)	Non-2R (32)	2R (16)	Non-2R (21)	Diagnosis	Genotype	Interaction
Age (years)	7.93 ± 1.91	8.75 ± 1.83	9.63 ± 1.63	9.38 ± 1.80	< 0.01	0.49	0.20
Gender (M/F)	11/4	28/4	11/5	10/11	0.003	0.37	0.14
IQ, mean (SD)	115.47 ± 13.31	120.38 ± 15.18	118.75 ± 12.57	120.67 ± 15.08	0.59	0.30	0.65
FD	0.10 ± 0.05	0.08 ± 0.05	0.08 ± 0.05	0.10 ± 0.05	0.55	0.91	0.11
Conners’ ratings							
Conduct problems	1.14 ± 0.36	1.10 ± 0.56	0.33 ± 0.35	0.42 ± 0.27	< 0.001	0.52	0.79
Learning problems	1.88 ± 0.74	1.87 ± 0.67	0.64 ± 0.63	0.68 ± 0.77	< 0.001	0.95	0.87
Psychosomatic	0.45 ± 0.41	0.40 ± 0.34	0.20 ± 0.24	0.14 ± 0.27	0.001	0.44	1
Anxiety	0.60 ± 0.42	0.42 ± 0.47	0.34 ± 0.31	0.30 ± 0.27	0.04	0.21	0.46
Impulsivity-hyperactivity	1.65 ± 0.62	1.48 ± 0.62	0.42 ± 0.47	0.46 ± 0.48	< 0.001	0.61	0.40
Hyperactivity index	1.56 ± 0.52	1.43 ± 0.54	0.42 ± 0.42	0.48 ± 0.39	< 0.001	0.74	0.37
IVA-CPT							
FRCQ	60.80 ± 24.56	61.50 ± 23.54	76.38 ± 24.68	80.62 ± 21.72	0.002	0.65	0.74
FAQ	65.33 ± 24.02	70.75 ± 22.81	88.38 ± 18.56	98.52 ± 17.92	< 0.001	0.11	0.63

IVA-CPT, integrated visual and auditory continuous performance testing. FRCQ, full-scale response control quotient; FAQ, full-scale attention quotient

### Data acquisition

All imaging data were acquired at the Department of Radiology. All participants were scanned on a GE Signa HDx 3.0 T scanner with an eight-channel phased-array head coil. During resting-state fMRI, the subjects were instructed to keep their eyes closed, relax their mind, and remain as still as possible without falling asleep. Plastic foam pads were used to minimize noise and the head movements of the subjects. Firstly, all subjects underwent a T2 weighted sequence scan to exclude any structural lesions in the brain. Then, resting-state functional MR images were obtained via an echo-planar imaging sequence (EPI; 31 axial slices, slice thickness = 4 mm, slice gap = 0.2 mm, repetition time (TR) = 2000 ms, echo time (TE) = 30 ms, flip angle = 90°, matrix size = 64 × 64, and field of view (FOV) = 192 × 192 mm^2^). The R-fMRI lasted eight minutes in total, and 240 volumes were obtained for each participant. Children were accompanied by a guardian to ensure their safety during the scanning process.

### Data preprocessing

The resting-state data were preprocessed using the toolbox for Data Processing and Analysis for Brain Imaging (DPABI 3.0; http://www.rfmri.org/dpabi) in Matlab 7.12.0 software (R2011a; https://cn.mathworks.com/products/matlab.html, Yan et al., 2016). In detail, the first ten volumes were discarded for scanner calibration, leaving 230 volumes. Slice timing and realignment were performed to correct the time differences between slices and head motion. In this study, any subjects with head motion > 3 mm (displacement) or 3° (rotation) were discarded. Subsequently, the functional images were spatially normalized to the EPI Montreal Neurological Institute (MNI) template (resampling voxel size = 3 × 3 × 3 mm^3^) to eliminate differences in the brain spatial structure of each subject. Further preprocessing included spatial smoothing with a 4-mm full width at half-maximum (FWHM) Gaussian kernel. Removal of linear trends and a temporal filter (0.01–0.1 Hz, to reduce low-frequency drifts and high-frequency physiological noise) were then applied. Finally, several sources of spurious variance, including 24 head motion parameters, white matter signal intensity, and cerebrospinal fluid signal intensity, were extracted and regressed from the data.

### Head motion

During preprocessing, four children with ADHD were excluded for having head motion > 3 mm (displacement) or 3° (rotation). In-scanner head motion during EPI acquisition was indexed by mean frame-wise displacement (FD) derived from Jenkinson’s relative root mean square algorithm (Jenkinson et al., 2002). Mean FD was used due to its consideration of voxel-wise differences in motion in its derivation (Yan et al., 2013). Participants with mean FDs exceeding one standard deviation (S.D.) above the sample mean (0.12 + 0.13 = 0.25 mm) were also excluded from the analysis. Using this criterion, nine participants (eight in the ADHD group and one in the healthy group) were excluded, yielding a final sample of 84 participants. There was no significant difference in mean FD between the ADHD (*n* = 47) and healthy control (*n* = 37) groups.

### Degree centrality analysis

Based on the preprocessing, DC calculations were performed using the GRaph thEoreTical Network Analysis (GRETNA) toolbox (http://www.nitrc.org/) in a voxel-wise manner with a threshold of *r* > 0.2, as previously described (Wang et al., 2015). Briefly, for each subject, Pearson correlation coefficients (*r*) were computed for all possible pairs of voxels, which resulted in an individual whole-brain functional connectivity matrix (Zuo et al., 2012). Finally, a degree centrality map for each individual was obtained and standardized to a *z*-score map using the Fisher *r*-to-*z* transformation.

Degree centrality can be affected by the connectivity distance between regions. Therefore, for further analysis, we subdivided the connections and associated degree centrality between all possible pairs of voxels into long-range (> 75 mm) and short-range (< 75 mm) categories according to their anatomical distance.

### Genotyping

For genotyping, DNA was extracted from 10 mL of peripheral venous blood followed by cell lysis and heme/protein precipitation. Genomic DNA was analyzed using TIANGEN (NO. DP304). Then, we genotyped the 48 bp VNTR in the Exon-3 DRD4 gene of each subject using polymerase chain reaction (PCR) and short tandem repeat (STR) methods. The primers used in this protocol are as follows: forward: 5’- GCG ACT ACG TGG TCT ACT CG-3’, reverse: 5’-AGG ACC CTC ATG GCC TTG-3’. PCR reactions were prepared in 25 μL volumes, each containing 12.5 μL KAPA2G Fast Multiplex Mix, 1 μL Forward primer (10 μm), 1 μL Reverse primer (10 μm), 1 μL Template DNA and 9.5 μL ddH2O. The samples were heated at 95◦C for 10 min and then cycled 40 times at 94◦C for 30 s, 55◦C for 30 s and 72◦C for 1 min, followed by 72◦C for 3 min with a final extension at 4◦C.

Then, the GS500LIZ standard sample and PCR products were mixed. Next, the atlas of STR was obtained using HIDI for electrophoresis. All participants were genotyped for the DRD4 48-bp repeat VNTR polymorphism.

### Statistical analyses

SPSS 22.0 software (SPSS Inc., Chicago, IL, USA) was used to conduct non-neuroimaging data analyses. A two-tailed independent-sample *t*-test was used for normally distributed variables. Non-normally distributed data were evaluated by Mann–Whitney *U* tests. Two-tailed *p*-values < 0.05 were considered statistically significant.

A voxel-wise two-way ANCOVA was performed separately to examine the main effects of diagnosis (ADHD vs. HC) and DRD4 genotype (2R vs. non-2R) and diagnosis-by-genotype interactions on DC maps, using age, gender and mean FD as covariates. The significance level was set at *p* < 0.05, corrected for multiple comparisons using the Gaussian random field (GRF) theory (voxel significance: *p* < 0.001, cluster significance: *p* < 0.05, two-tailed). Once regions showed the significant diagnosis-by-genotype interactions, two-sample t-test were performed to examine the connectivity differences between the genotype DRD4 2R and non-2R subjects in the ADHD and HC groups separately, with nuisance covariates including age, gender, IQ and mean FD.

Partial correlations were calculated to associate the DC of clusters, which showed significant *genotype* × *diagnosis* interaction effects, with the *Z*-scores of neuropsychological tests or clinical symptoms. These analyses were conducted separately for each subgroup and included the nuisance covariates of age, gender, IQand mean FD. Bonferroni corrections were performed, setting the significant *p*-value at 0.002 (= 0.05/(8 × 3), where 8 represents the number of neuropsychological tests and 3 represents the significant clusters of DC analyzed).

## Results

### Demographic and clinical characteristics

The remaining eligible participants were classified as being in the DRD4 2R group (ADHD: *n* = 15, twelve with the 2R/4R genotype and one each with 2R/2R, 2R/5R, or 2R/3R genotypes; HC: *n* = 16, fourteen with the 2R/4R genotype and two with 2R/2R) or the non-2R group (ADHD: *n* = 32, 28 with the 4R/4R genotype and four with the 4R/5R genotype; HC: *n* = 21, all with the 4R/4R genotype). The genotypes were in Hardy–Weinberg equilibrium (*p* > 0.05).

As shown in Table 1, there were no significant differences between genotypes in terms of their demographic factors. However, there were significant differences in gender and age distributions between the ADHD and HC groups (*p* < 0.05). Additionally, the ADHD group showing worse impulsivity or hyperactivity performance than the HC group (*p* < 0.05).

### DC analysis

**Fig. 1:**
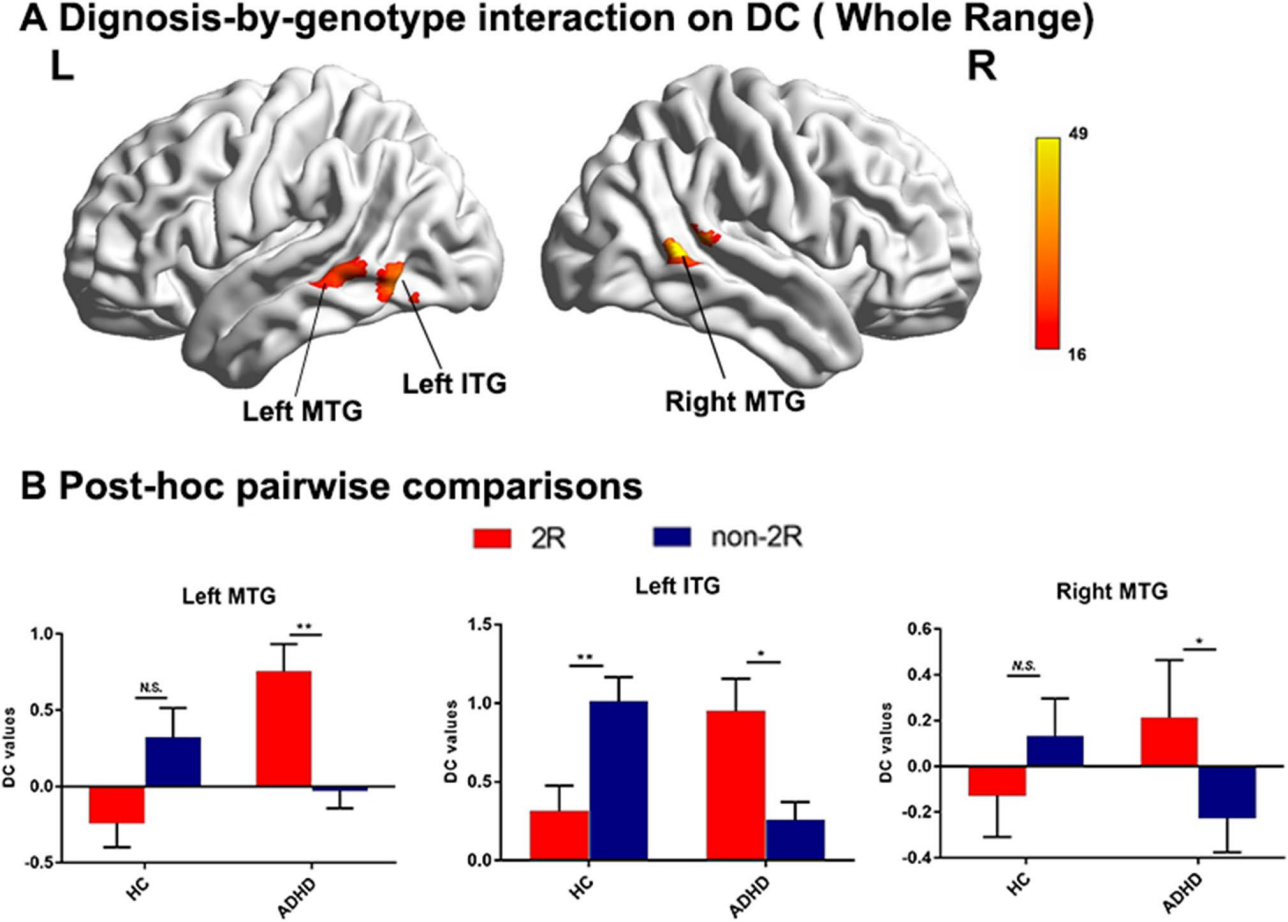
Diagnosis-by-genotype interactions on DC (Whole-Range). **A** Two-way ANCOVA revealed significant diagnosis by-genotype interactions on DC in the bilateral middle temporal gyrus (MTG), left inferior temporal gyrus (ITG). The color bar represents the statistical significance threshold (F-score). Multiple comparisons were conducted by Gaussian Random Field (GRF) theory (voxel significance: p < 0.001, cluster significance: p < 0.05). **B** The bar graphs depict post-hoc pairwise comparisons in the regions showing significant diagnosis-by-genotype interactions. The data were expressed as the mean (M) ± standard error (SE). DC, degree centrality; HC, healthy control; and ADHD, Attention-deficit/hyperactivity disorder. N.S, Non-significant. *p < 0.05; **p < 0.01

The whole-brain analysis of the whole-range degree centrality network revealed no significant genotype or diagnosis effects. However, significant *genotype* × *diagnosis* interactions on DC were found in the left inferior temporal gyrus (ITG) and bilateral middle temporal gyrus (MTG; GRF corrected *p* < 0.001, two-tailed; see Fig. 1A and Table 2). We then did the Post-hoc analysis for the genotype effect in ADHD and healthy controls separately. The analysis further revealed that the left ITG and bilateral MTG exhibited significantly higher DC values in the ADHD subjects with the DRD4 2R genotype (p < 0.05). Howerver, we observed significant lower DC values for the left ITG and a lower trend for bilateral MTG in the HC group with DRD4 2R (see Fig. 1B).

**Table 2:** Diagnosis-by-genotype interactions on degree centrality (DC)

		Cluster Size (voxels)		MNI coordinates (Peak)		
Cluster regions	BA		F value	X	Y	Z
Whole range						
Left ITG	37	19	32.02	-54	-63	-6
Left MTG	21	9	23.95	-66	-42	-3
Right MTG	21	21	49.02	63	-48	9
Short range						
Left MTG and ITG	21/37	59	30.28	-66	-42	-3
Left MTG	22	10	22.01	-57	-15	0
Right MTG	21	11	28.93	63	-48	9
Long range						
Left MTG	37	8	27.08	-54	-63	-6
Right MTG	21/22	17	43.84	63	-48	9
Right MFG	45	11	21.56	42	39	21
Left SPG	7	9	31.86	-30	-63	60

*ITG* inferior temporal gyrus, *MTG* middle temporal gyrus, *MFG* middle frontal gyrus, *SPG* superior parietal gyrus, *BA* Brodmann area, *MNI* Montreal Neurological Institute

**Fig. 2:**
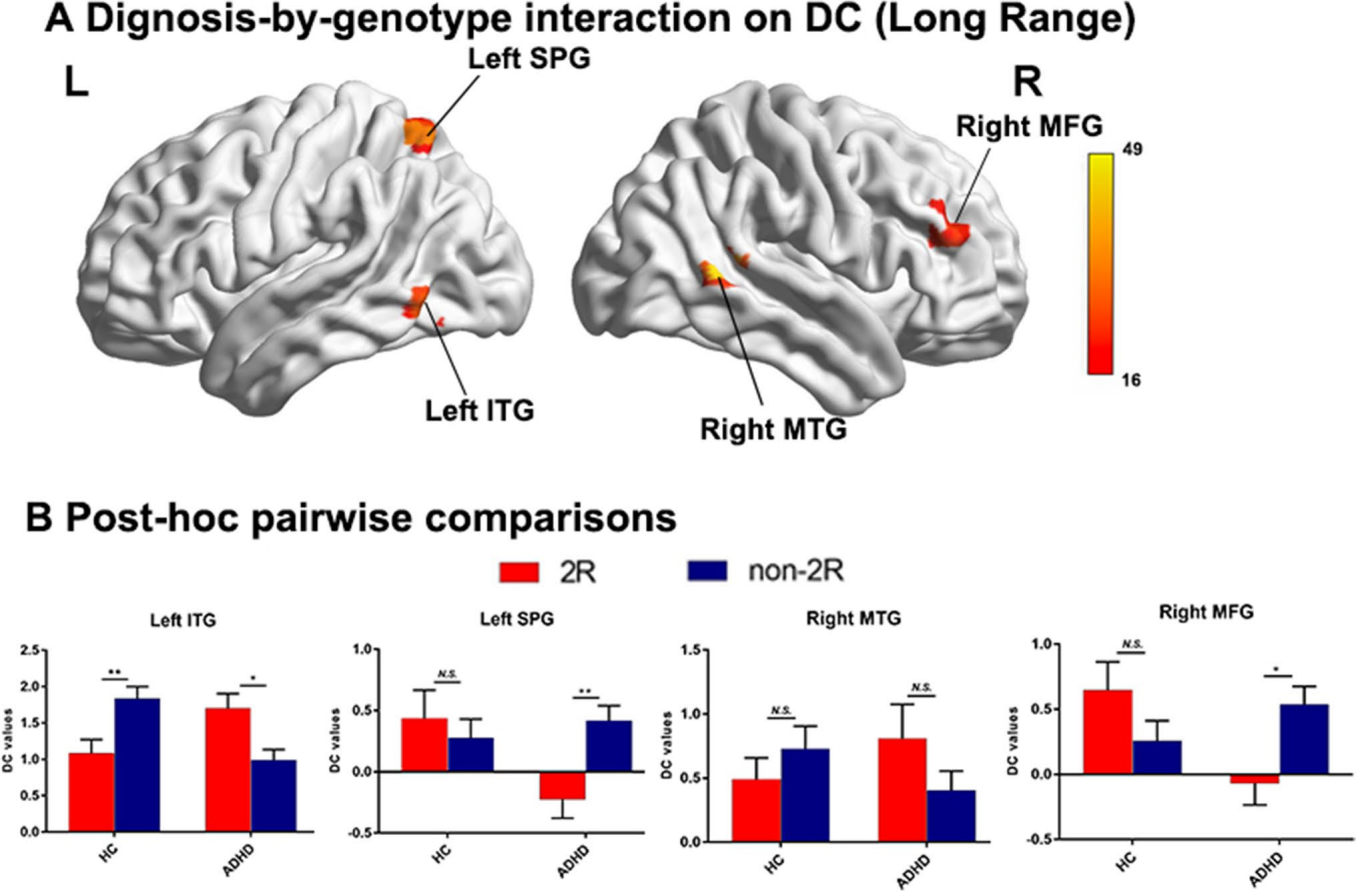
Diagnosis-by-genotype interactions on DC (Long-Range). **A** Two-way ANCOVA revealed significant diagnosis by-genotype interactions on DC in the left inferior temporal gyrus (ITG), right middle temporal gyrus (MTG), left superior parietal gyrus (SPG) and right middle frontal gyrus (MFG). The color bar represents the statistical significance threshold (F-score). Multiple comparisons were conducted by Gaussian Random Field (GRF) theory (voxel significance: p < 0.001, cluster significance: p < 0.05). **B** The bar graphs depict post-hoc pairwise comparisons in the regions showing significant diagnosis-by-genotype interactions. The data were expressed as the mean (M) ± standard error (SE). *DC* degree centrality, *HC* healthy control, *ADHD* attention-deficit/hyperactivity disorder. *N.S* non-significant. *p < 0.05; **p < 0.01

**Fig. 3:**
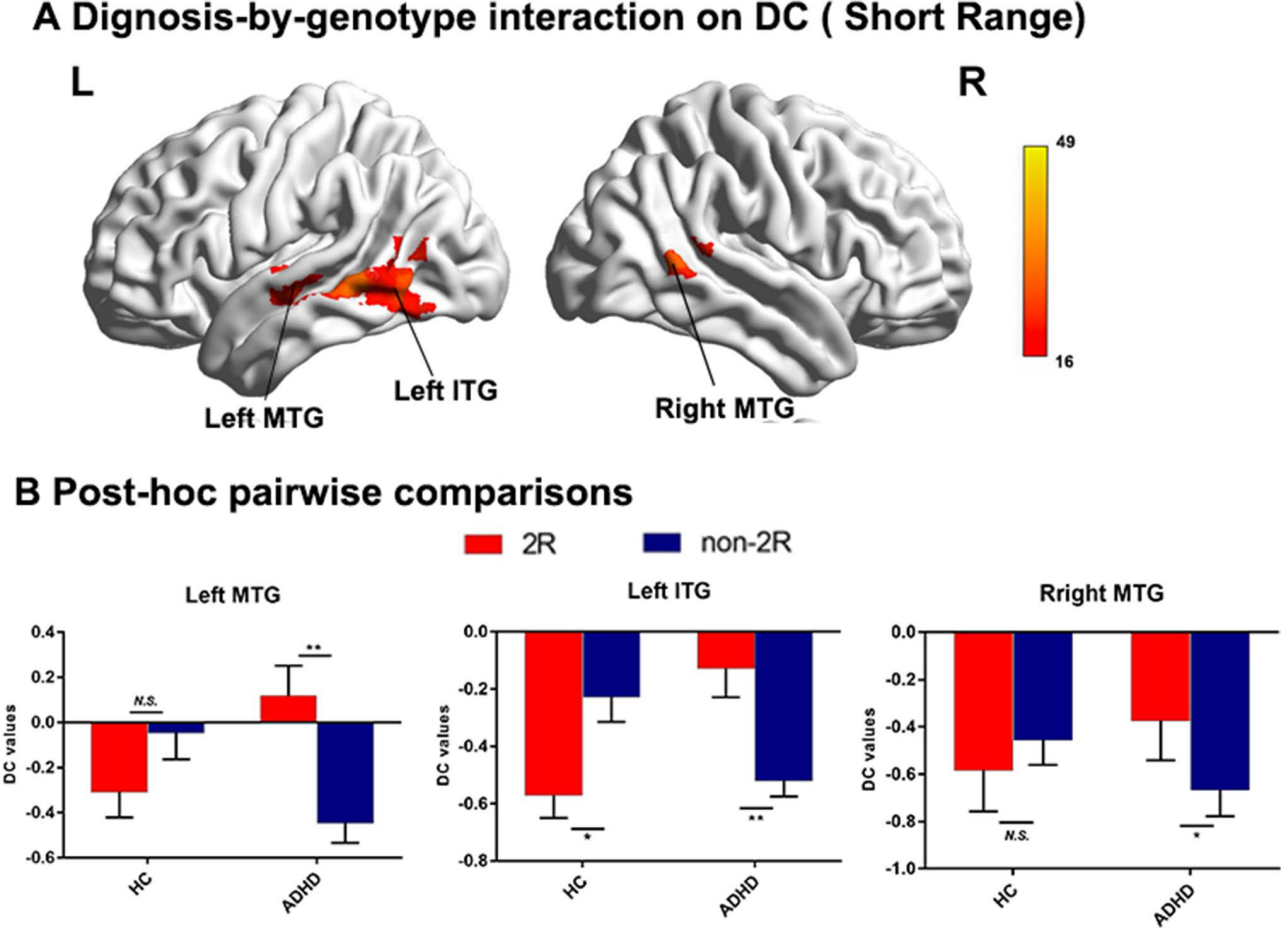
Diagnosis-by-genotype interactions on DC (Short-Range). **A** Two-way ANCOVA revealed significant diagnosis by-genotype interactions on DC in the left inferior temporal gyrus (ITG), bilateral middle temporal gyrus (MTG). The color bar represents the statistical significance threshold (F-score). Multiple comparisons were conducted by Gaussian Random Field (GRF) theory (voxel significance: p < 0.001, cluster significance: p < 0.05). **B** The bar graphs depict post-hoc pairwise comparisons in the regions showing significant diagnosis-by-genotype interactions. The data were expressed as the mean (M) ± standard error (SE). *DC* degree centrality, *HC* healthy control, *ADHD* attention-deficit/hyperactivity disorder. *N.S* non-significant. *p < 0.05; **p < 0.01

Since distance is a major potential confounding factor in the study of functional connectivity, we further subdivided the network into long- and short-range degree centrality networks (Liang et al., 2013). In addition to the similar results found for both short- and long-range connections (see Figs. 2 and 3), there were additional brain regions found in the long-range connection category, including the left superior parietal gyrus (SPG) and right middle frontal gyrus (MFG). Post-hoc analysis also confirmed that the genotypic effect was opposite in the ADHD and HC groups. The ADHD subjects with the genotype DRD4 2R showed significantly higher DC values in the left SPG and right MFG. In contrast, controls with 2R only showed a decrease in the trend in DC values.

### Correlational analysis

We found that the DC values in the left ITG were positively correlated with learning problems in the ADHD 2R group (p = 0.038). The DC values in the bilateral MTG were negatively correlated with full-scale response control quotient (FRCQ) in the ADHD non-2R group (p = 0.009 and p = 0.033). We also found correlations between the DC values in the left ITG and psychosomatic problems in the healthy controls 2R group (p = 0.03). However, these above correlational analyses did not pass the multiple corrections process.

## Discussion

Using resting-state fMRI and a network centrality approach, we found the first evidence of a significant interaction of DRD4 *diagnosis-by-genotype* on the brain functional architecture of the ITG and MTG. We found that, in the ADHD group, DC revealed genotype DRD4 2R-related increases in network centrality within the left ITG and bilateral MTG, and decreased long-range network centrality within the left SPG and right MFG. However, these genetic effects were opposite in healthy controls, although some of the results are not statistically significant. Despite, there was still have a certain trend. Our results reveal a divergent effect of the DRD4 genotype on functional connectivity in brain regions with relatively abundant DRD4 expression. As a result, the neuro-functional mechanisms underlying ADHD may differ according to the effect of the VNTR of the DAT1 gene.

Few studies have explored the interactive effect of DRD4 *genotype* × *diagnosis* in children with ADHD. A recent genetic imaging study examined the effect of DRD4 7R allele and ADHD interaction on brain structure. It found that prefrontal gyrification, which is associated with executive function, is reduced in ADHD subjects with the risk allele (Palaniyappan et al., 2019). Another fMRI study explored the genotype DRD4 2R allele and ADHD interaction effect on dynamic regional homogeneity (ReHo) and showed that the interaction effect was located in the superior parietal cluster (Kim et al., 2018). In the present study, we found a significant interaction effect in the fronto-parietal-temporal lobes, which was largely driven by altered DC in children with ADHD who carried the DRD4 2R allele.

The temporal lobe is mainly associated with the processing of sensory and emotional information (Aldhafeeri et al., 2012). Increased activity in the MTG prompts greater involvement in processing emotional stimuli (Gehricke et al., 2015). In addition, the part adjacent to the ITG is related to the identification and learning of two-dimensional graphic (Gross, 2008). Furthermore, the temporal lobe is an important part of the default mode network (DMN) and a large number of structural and functional studies have shown abnormal temporal lobes in ADHD subjects (Kobel et al., 2010). These findings suggest that the temporal lobe may play a key role in the pathophysiology of ADHD. In the present study, we observed genotype DRD4 2R-related increases in degree centrality on the left ITG and bilateral MTG in ADHD children. This indicates that the left ITG and bilateral MTG may act as key afferent or efferent hubs within the brain network, thereby maintaining information flow in ADHD subjects with the 2R allele. In genetic imaging research, a single photon emission-computed tomography (SPECT) study reported significantly higher perfusion in the MTGs of ADHD children with the 7R allele of the DRD4 gene than in those without the risk variant/genotype (Szobot et al., 2005). One explanation is that higher recruitment of this brain area might occur to compensate for a putative effect of risk alleles, probably determining dopaminergic dysfunction in that cerebral region. Consistent with these studies, the increased intrinsic network centrality within the bilateral MTG and left ITG in the ADHD 2R-present group may reflect that greater cognitive “effort” must be exerted by 2R-present subjects to achieve the same level of performance as ADHD non-2R subjects.

We also found a genotype DRD4 2R-related decrease in long-range degree centrality in the left superior parietal gyrus (SPG) and right middle frontal gyrus (MFG) in ADHD children but not in healthy children. This suggests a less prominent role of the MFG and SPG in coordinating the long-range functional brain network in ADHD subjects with the 2R allele. The prefrontal and parietal cortices possibly mediate more general meta-motor executive control functions, such as motor attention, conflict monitoring, and response selection. The prefrontal and parietal cortices are intricately connected to provide unified and tightly regulated attention functions (Arnsten & Rubia, 2012). Previous neuroimaging studies have shown that abnormal regional activity in the prefrontal and parietal cortices and hypoconnectivity of the fronto-parietal network (FPN) are involved in ADHD pathophysiology (Castellanos & Proal, 2012; F. Li et al., 2014; Tomasi & Volkow, 2012; Yang et al., 2011). The baseline hyperactivity of these sensory and motor-related cortices in ADHD patients may be due to an inability to suppress responsiveness to irrelevant sensory stimuli in the environment and may be associated with hyperactivity (Lee et al., 2005). A DRD4-related fMRI study reported that healthy DRD4 7R carriers showed reduced prefrontal brain activation during EF tasks, suggesting the 7R allele’s modulation of the sub-sensitivity of the DRD4 receptor (Gehricke et al., 2015). Using genetic neuroimaging, another study found a significant effect of diagnosis by DRD4 variant genotype on the superior parietal area, which was related to dysfunctional sustained and divided attention. One might speculate that the decreased DC of the MFG and SPG in the 2R group of ADHD children might result from sub-sensitive dopamine receptors. It is worth noting that the altered DC was limited in the long-range network. A balance between short- and long-range cortical interactions is essential for efficient cortical processing. Children's brain networks are dominated by short-distance connections, but as children mature, long-distance connections become dominant (Li et al., 2014). The present findings indicate that the brain networks of children with ADHD who carry the DRD4 2R allele may be underdeveloped.

It is also noteworthy that we observed the genetic effect to be opposite in healthy controls; however, only some of the results of the post-hoc analysis were statistically significant. A few genetic imaging studies have been performed in healthy children and adolescents. For example, a combined stimulus–response task study found that the 7R allele can influence both regional brain activation patterns and connectivity patterns between the neural networks of incompatibility and temporal processing. Another study observed that individuals with the 7R allele are more responsive to negative emotional stimuli than those with the 4R allele (Gehricke et al., 2015; Gilsbach et al., 2012). These results prove that there is a genetic effect of DRD4 on the specific task-related brain activities of healthy people. However, we failed to find any resting-state functional imaging studies related to the genotype DRD4 2R in healthy controls. Our findings provide preliminary evidence that the regulatory effects of DRD4 2R may differ, being more significant in ADHD subjects than in others.

Taken together, the present study focused on the interaction of DRD4 *diagnosis-by-genotype* on the network property of individuals with ADHD, which has been rarely studied. The major strength of this study was medicine-naïve patient samples and relatively strict head-motion correction. Only medicine-naïve patients were recruited to control for the confounding effects of previous pharmacological treatment. Previous studies have demonstrated that a psychostimulant drug will normalize the effects of DRD4 in PFC pyramidal neurons during cognitive tasks. In other words, the genetic effects of the DRD4 genes will be revealed mostly when the children are under the lowest putative dopamine levels, that is, at baseline. As mentioned previously, the DRD4 2R allele needs our further attention because of the difference in distribution among the ethnic population. In contrast to previous studies, we adopted the DC, which is a data-driven method without prior assumptions. Besides, it provides an approach that incorporates analysis of the whole-brain complex network at once and can overcome the limitation of exploring only some sub-networks in seed-based FC and independent component analysis.

Several limitations in the present study need to be mentioned. First, the sample size of this ANCOVA analysis (DRD4 2R group (ADHD: *n* = 15; HC: *n* = 16) and the non-2R group (ADHD: *n* = 32; HC: *n* = 21)) was relatively small, which restrict the statistical power. Larger sample size and multicenter studies will help us to fully understand the mechanisms of genetic risk underlying ADHD. Second, the interactive analyses revealed no significant main effect of diagnosis on brain network centrality. In contrast, previous neuroimaging studies have observed much abnormal activity in certain brain regions of ADHD subjects, such as the right inferior frontal gyrus (rIFG), sensorimotor cortex, and anterior cingulate cortex (Alonso Bde et al., 2014; Lei et al., 2015; Silk et al., 2016). This might be attributable to there being a greater proportion of boys, and lower ages, in the ADHD group of the present study than in controls. A larger and more reasonable sample distribution should be used in future studies to provide further validation of this relationship. Third, it is not comprehensive to assess the effect of a single candidate gene in a polygenic disorder. Previous studies have revealed that the brain structure and function of ADHD subjects can be influenced by multiple gene variants, especially dopamine-related genes (Klein et al., 2017). Thus, further genetic imaging studies combining a set of risk variants and examining more complex gene–gene interactions on brain network topology are necessary to obtain sufficient information. Fourth, ADHD is a complex heterogeneous disease comprising different subtypes (inattention, hyperactive, and combined) and comorbidity with psychiatric conditions, and we did not conduct further subtype analyses due to the small sample size. Finally, the use of genetic imaging methods in a cross-sectional cohort study provides results that are inherently observational and correlational. Therefore, there is also a need to combine the growing evidence of genetic anomalies in ADHD with measures of brain dysfunction in longitudinal studies to determine whether the brain abnormalities change throughout the life cycle (Yadav et al., 2021).

## Conclusions

In the current study, we found that the DRD4 2R allele mainly affects the connectivity of the temporal and frontoparietal lobes, which involved emotional and attentional functions. Our findings also suggested that the DRD4 genotype differently modulates the functional integration of the brain networks of children with ADHD and healthy controls. This helps us to understand how the overall composition of the brain network is reorganized in response to DRD4 variants in ADHD subjects. And the neuroimaging findings associated with the DRD4 provide new insights into the heterogeneity of ADHD.

## Data Availability

The raw data supporting the conclusions of this article will be made available by the authors, without undue reservation.
